# A questionnaire study of injections prescribed and dispensed for patients diagnosed with mild/moderate community-acquired pneumonia in Mongolia

**DOI:** 10.7717/peerj.1375

**Published:** 2015-11-26

**Authors:** Gereltuya Dorj, Delia Hendrie, Richard W. Parsons, Bruce Sunderland

**Affiliations:** 1 School of Pharmacy and Biomedicine, Mongolian National University of Medical Sciences, Ulaanbaatar, Mongolia; 2 School of Public Health, Curtin University of Technology, Perth, Western Australia, Australia; 3 School of Pharmacy, Curtin University, Perth, Western Australia, Australia

**Keywords:** Injections, Pneumonia, Prescribing, Dispensing, Parenteral medication, Developing country, Mongolia

## Abstract

**Purpose.** The study aimed to determine the extent of and factors influencing the prescribing of injections for the treatment of mild/moderate community acquired pneumonia (CAP) in Mongolia.

**Methods.** Questionnaires were developed and administered to medication providers (34 Pharmacists, 27 pharmacy technicians) and prescribers (22 general doctors and 49 medical specialists) working in Mongolia.

**Results.** Cefalosporins were prescribed for patients with mild pneumonia and doctors tended to prescribe injectable cefalosporins (cefazolin) rather than oral dosage forms. This was supported by the questionnaire study with pharmacists and pharmacy technicians. Additionally, 23 pharmacists and pharmacy technicians indicated that OTC injectable cefalosporins (37.7%) and injectable aminopenicillins (33,9%) were frequently sold by pharmacies for the treatment of mild/moderate CAP. Doctors and particularly pharmacists in the questionnaire studies indicated choosing an injection was to avoid non-compliance problems.

**Conclusion.** High levels of injectable prescribing of antibiotics were found in non-hospitalized patients with CAP in Mongolia. This prevalence level indicated that inappropriate injection prescribing is a public health hazard for Mongolia and requires consideration by the appropriate authorities.

## Introduction

Medicines are commonly administered by injection in healthcare settings for the prevention, diagnosis, and treatment of various illnesses. Unsafe injection practices including the re-use of equipment without sterilization has been associated with a wide variety of procedures and settings and puts patients and healthcare providers at risk of infectious and non-infectious adverse events (World Health Organization, The SIGN Alliance, 2001).

The Global Burden of Disease project (World Health Organization (WHO)) conducted a literature review and found that the annual number of single injections administered per person ranged from 1.7 to 11.3 per year (Hutin, Hauri & Armstrong, 2003). A systematic review of studies from 13 developing countries regarding injection use and safety reported that for eight of those countries, 25–96% of outpatient visits resulted in at least one injection, and for five countries a majority of the administered injections was unnecessary (Simonsen et al., 1999). An assessment of injection practices in Mongolia showed a high injection frequency rate; reporting an average of 13 injections per person per year amongst a small sample of 65 participants and the most common reason for an injection was community-acquired pneumonia (CAP) (Logez et al., 2004).

CAP accounts for 95% of all pneumonia cases in the world among children aged less than five years of age (Wardlaw, Johansson & Hodge, 2006). In a prospective study in five countries of factors of CAP caused by bacteraemic pneumococcal disease, death rates ranged from 6% in Canada to 20% in the USA, 13% in the UK and 8% in Sweden (Kalin et al., 2000). The mortality rate from CAP for children aged less than five was 34.4% in 2011 in Mongolia and was the second most common reason for all hospitalizations in 2011 amongst children (46%) (Ariuntuya et al., 2011). The general consensus is that pneumonia should be treated with antibiotics and for those with mild/moderate CAP oral administrationis appropriate (World Health Organization, 2005; Antibiotic Expert Group, 2010).

The Mongolian standard treatment guidelines (STGs) for adult and paediatric patients are available and widely accessible in the Mongolian language. For adult patients, the STGs recommend oral amoxicillin (ampicillin) 500 mg every 6 h or erythromycin 500 mg every 6 h (Batsereedene, Naidansuren & Oyunbileg, 2003). The treatment standard for paediatric pneumonia is classified into infants and children aged up to five years old. The STGs for infant patients diagnosed with mild/moderate CAP recommends benzylpenicillin, aminoglycoside (gentamicin) injection. Treatment of children aged up to five years old should include semi-synthetic penicillin (50 mg/kg/4 times) plus gentamicin (7.5 mg/kg/once)-injection and other options include cephalosporin generations II–III. In addition, any of the following could be prescribed for children aged up to five years can be prescribed: salbutamol, euphyllin, epinephrine, prednisolone, dexamethasone, vitamin C, A or E, if considered to be necessary.

The reasons for prescribing parenteral medications in developing countries include socio-cultural, economic and structural factors. The belief in injections as a strong tool for restoring and maintaining health is mutually supported by health professionals and community members in many developing countries (Berild et al., 2002). Previous studies have indicated poor knowledge of the associated risks and burden of unsafe and unnecessary injection practices and easy access to parenteral medication contributes to the popularity of injections in developing countries (Birungi, 1998; Lakshman & Nichter, 2000; Reeler, 2000).

## Objective

The study aimed to determine the extent of, and factors influencing the prescribing of injections for the treatment of patients diagnosed with mild/moderate CAP in Mongolia.

## Materials and Methods

Two questionnaires were developed using a WHO/SIGN guide (Hulscher, Grol & van der Meer, 2010; World Health Organization, The SIGN Alliance, 2001). This guide included information relevant to investigation of injection practices, their determinants and their consequences. In addition, face validation of both questionnaires was performed with pharmacy academics and practitioners from Australia and Mongolia, to ensure the validity of questions exploring doctors’ and pharmacist’s practises of prescribing, dispensing and supplying injections for the treatment of CAP in Mongolia. Content validation was carried out with three family group practitioners and two specialists practising in a conveniently selected large central hospital (for the doctor questionnaire). The one for pharmacists and pharmacy technicians was tested with two pharmacists and two pharmacy technicians working in randomly selected pharmacies (representing the pharmacists). The purpose of the piloting phase was to ensure that the questions were clear, and achieved the objectives of the study. In addition, it was important that the instrument could be completed in a reasonable amount of time. Following their feedback and discussion with local professionals, the wording and order of some the questions were modified. The responses collected during the pilot studies were not used for further analyses. Respondents could choose more than one option in questions regarding the prescribing and dispensing practice of antibiotics and non-antibiotics for the treatment of mild/moderate CAP.

### Selection of study sites

As recommended in the WHO guide (World Health Organization, The SIGN Alliance, 2001), three large urban districts (based on population size), two sub-urban districts (Nalaikh, Baganuur) located in Ulaanbaatar known to be representative of all socioeconomic groups in Mongolia were selected. There are three public central hospitals, eight specialized centres, nine district hospitals, six private hospitals and 126 Family Group Practices (FGPs) located in Ulaanbaatar (Information and Technology Department of Ulaanbaatar, 2013). Selection of health facilities was done purposively based on their location and accessibility. For the study, three public central hospitals in large districts, three district hospitals in urban and two district hospitals in sub-urban districts, three private hospitals in sub-urban districts and 20 FGPs located in both large and small (sub-urban) districts were purposively selected.

A purposive sample of forty community pharmacies was selected from the five districts to represent a range of pharmacies based on their size, accessibility and distance from clinics, following discussions with local professionals, and ensuring that no particular type of pharmacy was excluded. These included 12 of the 22 pharmacies consenting to a previous study and a further 28 pharmacies were approached (Dorj et al., 2013). In respect to their location, 25 community pharmacies were located in three large districts and 15 were in two sub-urban districts.

Doctors working in the above-mentioned health facilities were randomly selected from the list of actively working employees, provided by human resource offices in the selected sites. Pharmacists and pharmacy technicians were conveniently selected from the pharmacies.

### Administration of the questionnaires

After obtaining verbal consent, a self-administered questionnaire was distributed by hand to eligible participants (pharmacist and/or pharmacy technician, doctor and/or specialist) working in community pharmacies or hospitals in urban and sub-urban districts of Ulaanbaatar, Mongolia.

In order to improve the response rate, the survey was issued in the early mornings or when the participants were able to focus on the survey. No more than two respondents were selected from the same pharmacy or clinic and where there were two, they were a pharmacist and a pharmacy technician or a doctor and a specialist. Respondents completed the questionnaire independently, if there were more than one respondent at the same setting. The questionnaires were collected from pharmacies and clinics by the researcher.

### Data analysis

Standard descriptive statistics were used to summarize demographic data and responses to the questionnaires. Questions regarding the frequency of dispensed/prescribed medicines for the treatment of CAP were answered using a five-point Likert scale ranging from never to always. For some analyses, the responses were condensed into three categories (never/rarely, sometimes, and often/always). Responses using Likert scales that ranged from strongly agree to strongly disagree were similarly formed into two groups, strongly agree/agree, and disagree/strongly disagree. For other analyses, the Likert scale responses were coded from one to five, so that mean scores and standard deviations could be calculated and used to compare differences between groups.

The mean values of responses measured on a Likert scale may be assumed to be normally distributed, as the number of respondents was large (>30) in each group, pharmacy and pharmacy technicians-(61), doctors-(71) (Central Limit Theorem) (Lumley et al., 2002).

Comparisons between groups based on route of administration (injection versus oral/other), setting (private versus public) and professional level (pharmacist versus pharmacy technician) were performed using the Chi-square test, logistic regression or Kruskal–Wallis test with a pairwise comparison as appropriate. A *p* value of <0.05 was taken as indicating a statistically significant difference.

### Ethical considerations and confidentiality

The study protocol was approved by the Human Research Ethics Committee, Curtin University, Western Australia (PH-11-2010). The questionnaire study was completed during January until May, 2010.

### Data accessibility and sharing

Extra data are available by emailing the corresponding author (GD).

## Results

Questionnaires were distributed to 45 pharmacists and 35 pharmacy technicians (*n* = 80). An overall response of 61 (76.3%) completed survey forms were returned from the ‘pharmacy group.’ Pharmacists (11) and pharmacy technicians (8) who did not consent were working in pharmacies located in the large districts. The refusal was due to busy workload and unwillingness to participate.

A majority of respondents was female (77.0%), and most were aged between 31 and 50 years. A little over half were pharmacists (55.7%), and most respondents had been working for one to five years (65.6%). The study included one pharmacist, and/or pharmacy technician from each pharmacy and accordingly they were visited in their working area. Where the two were at the same pharmacy, they completed the questionnaire independently.

In addition, questionnaires were distributed to 80 doctors of whom 35 were general practitioners (GPs) and 45 were medical specialists. The response rate was 88.8% (71/80) and 83.1% were female doctors, which is comparable to the gender distribution of all doctors in Mongolia (79.1%) (Ariuntuya et al., 2011). Most respondents were working in public hospitals and about 70% were specialists. A majority was over 30 years of age (63.4%) with a monthly income of over 300.000 MNT (Mongolian National Tugrug, currency, 100 USD is equivalent to 130,000 MNT at the time of study) (Table 1).

**Table 1:** Demographic characteristics of respondents.

Variable^a^	Category		Pharmacists and pharmacy technicians (*N* = 61) *n* (%)	Doctors (*N* = 71) *n* (%)
Age (years)	20–30		22 (36.1)	26 (36.6)
	31–50		23 (37.7)	45 (63.4)^c^
	≥51		16 (26.2)	
Gender	Male		14 (23.0)	12 (16.9)
	Female		47 (77.0)	59 (83.1)
Practice setting	Owner	Public hospital	12 (19.7)	54 (76.1)
	Employee	Private setting (including FGPs and others)	49 (80.3)	17 (23.9)
Professional level	Pharmacist	General doctor	34 (55.7)	22 (31.0)
	Pharmacy technician	Specialist	27 (44.3)	49 (69.0)
Years of working experience	1–5		40 (65.6)	34 (47.9)
	6–10		11 (18.0)	12 (16.9)
	≥11		10 (16.4)	25 (35.2)
Monthly income (MNT)^b^	90.000–200.000		9 (15.0)	12 (17.1)
	201.000–300.000		13 (21.7)	28 (40.0)
	≥301.000–400.000		23 (38.3)	30 (42.9)^d^
	≥401.000		15 (25.0)	

**Notes.**

a Some responses were missing for each category.

b Mongolian National Tugrug, (MNT), currency, 100 USD was equivalent to 130,000 MNT at the time of study.

c The number represents respondents aged ≥31 years old.

d The number represents respondents with a monthly income of ≥301.000 MNT and higher.

Pharmacists and pharmacy technicians reported that injections were prescribed and dispensed for the treatment of mild/moderate CAP. Commonly prescribed antibiotics that were dispensed were ampicillin injections (52.5%), injectable cefalosporins (75.4%) and injectable quinolones (55.7%). These were more frequently dispensed than the oral forms. Oral macrolides (50.8%) were dispensed more frequently than an injection (Table 2).

**Table 2:** Questionnaire percentage frequencies of antibiotics dispensed with prescription or provided OTC for treatment of CAP, orally and injection by pharmacists and pharmacy technicians (*N* = 61).

ATC classification^a^	With prescription^b^			Provided OTC^b^		
	Never/rarely *n* (<10%)	Sometimes *n* (11–40%)^b^	Often/always *n* (>40%)	Never/rarely *n* (<10%)	Sometimes *n* (11–40%)^b^	Often/always *n* (>40%)
Aminopenicillins, oral	77 (31.6)	70 (28.7)	97 (39.8)	79 (32.4)	73 (29.9)	92 (37.7)
Aminopenicillins, injection	55 (30.1)	51 (27.9)	77 (27.9)	77 (42.1)	44 (24.0)	62 (33.9)
Quinolone, oral	53 (43.4)	28 (23.0)	41 (33.6)	67 (54.9)	30 (24.6)	25 (20.5)
Quinolone, injection	13 (21.3)	14 (23.0)	34 (55.7)	30 (49.2)	13 (21.3)	18 (29.5)
Cefalosporin, oral	16 (26.2)	15 (24.6)	30 (49.2)	28 (45.9)	14 (23.0)	19 (31.1)
Cefalosporin, injection	7 (1.5)	8 (13.1)	46 (75.4)	28 (45.9)	10 (16.4)	23 (37.7)
Macrolides, oral	40 (21.9)	50 (27.3)	93 (50.8)	77 (42.1)	53 (29.0)	53 (29.0)
Macrolides, injection	131 (71.6)	26 (14.2)	26 (14.2)	134 (73.2)	29 (15.8)	20 (10.9)
Tetracycline, oral	103 (84.4)	16 (13.1)	3 (2.5)	91 (74.6)	19 (15.6)	12 (9.8)
Sulfonamid, oral	18 (29.5)	19 (31.1)	24 (39.3)	17 (27.9)	18 (29.5)	26 (42.6)

**Notes.**

a Some responses were missing for each category.

b Respondents could choose more than option.

According to the current regulations, only qualified medical doctors can prescribe prescription medicines approved by the order of the Minister of Health to patients (Ministry of Health Mongolia, 2010). However, the provision of over the counter (OTC) medicines, including injections occurs in Mongolian pharmacies (Margaret, 2011; Tsolmongerel et al., 2013). Pharmacists and pharmacy technicians were asked to indicate their practice of providing OTC antibiotics for the treatment of CAP. Most reported they never or rarely provided OTC medicines (65.0%); on the other hand 13 pharmacists and pharmacy technicians (21.7%) provided OTC antibiotics sometimes. However, when presented with questions related to medicines that were provided OTC, the respondents indicated a higher frequency of commonly provided OTC antibiotics, for example: injectable penicillins with extended spectrum (e.g., ampicillin) (36.1%). Additionally, respondents indicated that OTC injectable (37.7%) and oral cefalosporins (31.1%) were frequently provided. In contrast, injectable macrolides were less frequently provided (10.9%) (Table 2).

Other medicines dispensed with a prescription for the treatment of CAP included mucolytics (50.8%), oral vitamins (38.7%) and oral antihistamines (21%). Additionally, injectable corticosteroids (29.5%) and injectable xanthins (22.0%) were frequently dispensed non-antibiotics (Table 3). There was also some provision of oral or injectable non-antibiotic OTC medicines from pharmacies with regards to corticosteroids (about 10%) and pyrazolones (13%). Again, similar to the medicines dispensed with a prescription, the most common OTC medicines provided for treatment of CAP were oral vitamins and oral xanthins (Table 3).

**Table 3:** Frequencies of non-antibiotic medicines dispensed with prescription and provided OTC for treatment of CAP, by pharmacists and pharmacy technicians (*N* = 61).

Other medicines^a^	With prescription^b^			Provided OTC^b^		
	Never/rarely *n* (<10%)	Sometimes *n* (11–40%)	Often/always *n* (>40%)	Never/rarely *n* (<10%)	Sometimes *n* (11–40%)	Often/always *n* (>40%)
Corticosteroid, oral	31 (50.8)	19 (31.1)	11 (18.0)	35 (59.3)	16 (27.1)	8 (13.6)
Corticosteroid, injection	19 (31.1)	24 (39.3)	18 (29.5)	28 (48.3)	24 (41.4)	6 (10.3)
Vitamin, oral	31 (26.1)	42 (35.3)	46 (38.7)	28 (23.7)	30 (25.4)	60 (50.8)
Vitamin, injection	88 (49.4)	56 (31.5)	34 (19.1)	97 (55.1)	42 (23.9)	37 (21.0)
Antihistamin, oral	58 (48.7)	36 (30.3)	25 (21.0)	63 (53.4)	24 (20.3)	31 (26.3)
Antihistamin, injection	37 (62.7)	15 (25.4)	7 (11.9)	41 (70.7)	11 (19.0)	6 (10.3)
Xanthin,^c^ oral	18 (30.0)	25 (41.7)	17 (28.3)	21 (35.0)	15 (25.0)	24 (40.0)
Xanthin, injection	27 (45.8)	19 (32.2)	13 (22.0)	34 (57.6)	12 (20.3)	13 (22.0)
Pyrazolone, oral	45 (76.3)	7 (11.9)	7 (11.9)	35 (60.3)	13 (22.4)	10 (17.2)
Pyrazolone, injection	44 (75.9)	9 (15.5)	5 (8.6)	37 (63.8)	13 (22.4)	8 (13.8)

**Notes.**

a Some responses were missing for each category.

b Respondents could choose more than option.

c Xanthin is euphyllin.

In order to gain an understanding the underlying reasons for prescribing and providing OTC injections, respondents were asked to indicate the extent to which they agreed with issues that influenced their provision practice of OTC medicines (Table 4).

**Table 4:** Percentage frequencies of characteristics that influence the practice of providing medicines and prescribing for the treatment of CAP.

Characteristic^a^	Pharmacists and pharmacy technicians (*N* = 61)			Doctors (*N* = 71)		
	D/SD *n* (%)	SA/A *n* (%)	NR *n* (%)	Never/rarely *n* (<10%)	Sometimes *n* (11–40%)	Often/always *n* (>40%)
The clinical effect of injections is more potent than oral medicines’	14 (24.1)	40 (68.9)	4 (6.9)	16 (22.5)	24 (33.8)	31 (43.7)
The pharmaceutical quality of injections is better than tablets/capsules	17 (29.3)	34 (58.6)	7 (12.1)	15 (21.1)	27 (38.0)	29 (40.8)
Adverse events occur with oral drugs more than with injections	32 (55.2)	16 (27.6)	10 (17.2)	39 (54.9)	22 (31.0)	10 (14.1)
The dosage form of injection is chosen for better compliance of a patient	9 (15.8)	39 (68.4)	9 (15.8)	34 (47.9)	6 (36.6)	11 (15.5)
The injection requires new syringes and needles	8 (13.8)	48 (82.8)	2 (3.4)	2 (2.8)	7 (9.9)	62 (87.3)
Training promotes more about treatment with an injection than oral medicines	38 (65.5)	12 (20.7)	8 (13.8)	52 (73.2)	15 (21.1)	4 (5.6)
There is lot of advertisement about injections by drug companies	38 (65.5)	11 (19.0)	9 (15.5)	35 (49.3)	27 (38.0)	9 (12.7)
Cost of treatment by oral medicines is more than the treatment cost with injections (including cost of syringes and needles)	31 (56.9)	21 (36.2)	4 (6.9)	45 (63.4)	11 (15.5)	15 (21.1)
If patients are prescribed an injection, they are required to visit a pharmacy/hospital several times	32 (55.1)	23 (39.7)	3 (5.2)	8 (11.2)	19 (26.8)	44 (62.0)
Patients prefer to use tablets rather than injection	35 (60.3)	17 (29.3)	6 (10.3)	30 (42.3)	24 (33.8)	17 (23.9)
When dispensing injections, patient’s age, gender are important	10 (17.2)	44 (75.9)	4 (6.9)	8 (11.3)	16 (22.5)	47 (66.2)
Injection is chosen if patient had severe CAP	10 (17.2)	46 (79.3)	2 (3.4)	16 (22.5)	21 (29.6)	34 (47.9)

**Notes.**

SA Strongly agree A Agree D Disagree SD Strongly disagree NR No response

a Some responses were missing for each category.

Most respondents commonly reported that injections were provided if patients had severe CAP (pharmacists and pharmacy technicians (79.3%), doctors (47.9%)). A majority of pharmacists and pharmacy technicians strongly agreed that injections were provided OTC to achieve better patient compliance with treatment (68.4%), however this reason was supported by only eleven doctors (15%). Male doctors tended to agree more than females with injections improving patient compliance with a treatment regimen (*p* = 0.011, (male *M* = 3.0, STD = 1.17, f: *M* = 2.0, STD = 0.9)).

Amongst respondents, a fairly high proportion of pharmacists and pharmacy technicians (69%) and 44% of doctors specified that the clinical effect of injections was better than oral medicines. No significant relationship was observed between pharmacists and pharmacy technicians (*p* = 0.59). Additionally, the proportion of pharmacists supporting the idea that medication outcomes from injections were better than tablets or capsules tended to be greater (62.5%) than pharmacy technicians (53.8%), yet it was not statistically significant: (*p* = 0.51). Most pharmacists, including pharmacy technicians (59%) and some doctors (41%) stated that the pharmaceutical quality of injections was better than oral medicines.

About 70% of pharmacists including pharmacy technicians did not support the view that adverse effects occurred more with oral medications than with therapeutic injections. Similarly, only 14% of doctors agreed that the prevalence of side effects was lower with injections than with oral medicines. In addition, 21 pharmacists and pharmacy technicians (36.2%) strongly agreed/agreed that the cost of treatment with injections was higher than with oral medicines. However, only 15 doctors (21%) supported this.

A similar number of doctors (29%) and pharmacists plus pharmacy technicians (23.9%) supported the statement that patients preferred oral medicines over injections (Table 4).

## Discussion

This study was undertaken to quantitate the levels injection prescribing by doctors and provision OTC by community pharmacies from their perspectives, for the treatment of mild/moderate CAP in Mongolia. Importantly it has identified some factors influencing the prescribing of injections which could lead to interventions to improve the underlying quality of prescribing and reduce that public health hazards associated with the administration of injections.

Pharmacists and pharmacy technicians indicated that only 21.7% provided OTC antibiotics sometimes. However, the frequency of OTC injectable antibiotics provided was reported as higher elsewhere in the same questionnaire, possibly better indicating real practice when compared with previous findings (Dorj, Sunderland & Hendrie, 2014). Oral and injectable aminopenicillins (ampicillin or amoxicillin) were commonly prescribed (Dorj et al., 2013) and this was confirmed by the questionnaire study with pharmacists, including pharmacy technicians and doctors. This practice was in compliance with the guidelines (Agvaandorj et al., 2005). However, the guidelines allow for only oral aminopenicillins for adult and pediatric patients yet 27.9% of prescribing were injections. Pharmacists and pharmacy technicians indicated a higher likelihood of supplying OTC oral or injectable aminopenicillins (37.7% versus 33.9%). Cefalosporins were prescribed for patients with mild pneumonia and doctors tended to prescribe injectable cefalosporins (cefazolin) rather than oral, and this was supported by the questionnaire study with pharmacists and pharmacy technicians. OTC sales of cefazolin were also reported in the questionnaire study, but the pharmacists and pharmacy technicians did not indicate any preference for either of the dosage forms.

The range of non-antibiotic medicines prescribed for patients with mild/moderate CAP included vitamins, mucolytics, corticosteroids and antihistamines (Dorj et al., 2013). However, the prescription results showed that only four injectable vitamins were prescribed. This is in contrast to the questionnaire studies where doctors indicated a higher frequency of prescribed injectable vitamins (25.8%) often/always for patients with mild CAP. The practice of selling by pharmacies of injectable vitamins OTC was also found to be at a similar level (21%). The practice of providing vitamin injections OTC is not consistent with current STGs. Detailed results from a previously reported prescription analysis (Dorj et al., 2013) and these questionnaire studies showed that vitamin C was frequently prescribed and dispensed for the treatment of mild/moderate CAP. This could reflect a low fresh food intake containing necessary vitamins, leading to micronutrient deficiencies in Mongolia (Kachondham et al., 1992; Lander et al., 2008). A Cochrane review of five trials suggested vitamin C was beneficial in both prevention and treatment of pneumonia. However, caution must be exercised with generalizations made from trials owing to the conditions in which the trials were conducted. But for patients who have low plasma vitamin C levels, intake of vitamin C would be beneficial (Hemilä & Louhiala, 2007).

The questionnaire studies with doctors and pharmacists including pharmacy technicians indicated that they chose an injection if the patient was severely ill. This perspective is consistent with guidelines and several findings from other countries (Chowdhury et al., 2011; McIntosh, 2002; Vong et al., 2005). Likewise, considering the period of study (cold winter) and risk of deterioration of the patient, this practice may reflect clinical concern. However, choosing an injection for adult patients with mild/moderate CAP is non-compliant with current STGs (Agvaandorj et al., 2008; Mongolian National Center for Standardization and Metrology, 2008).

Financial considerations are another important reason why injections were preferred by prescribers and providers. In this study most respondents acknowledged that the cost of treatment with injections was higher than with oral medicines. Economic incentives from prescribing an injection were reported in a previous study where 19% of high rate injection prescribers admitted having economic incentives for prescribing injections in Iran (Ismaeilzadeh, Nikfar & Rahimi, 2006). The large number of doctors on a population basis practising in Mongolia maybe a potential factor for doctors to seek additional income sources.

Additionally, one of the factors that contributed to the inappropriate use of injections in developing countries has been the prescriber’s perception that patients preferred them (Hutin, Hauri & Armstrong, 2003; McIntosh, 2002; Vong et al., 2005; Ismaeilzadeh, Nikfar & Rahimi, 2006; Hadiyono et al., 1996). In this study, approximately 24% of doctors and 29% of pharmacists, plus pharmacy technicians strongly supported the notion that patients often/always preferred oral medications. This is contrasted with findings that only 16% of community members always/often expected injections to be prescribed (Srinivasan, 2004). A previous research study that investigated maternal perception of mild pneumonia in an outpatient clinic found that 40% of mothers stated doctors should give their child at least one injection. However, the generalization of that report to a larger population might be questionable, due to a small number and poor understanding of the participants (*n* = 50) (Malik Kundi et al., 1993). Other literature has confirmed that injections were often not preferred by patients, when they were advised about the clinical efficacy and potential risks associated with unsafe injection practices (Hadiyono et al., 1996). Health workers in developing countries believed that patient’s compliance was better with injections than with oral medication (Michelle, 2004; AnneLoes van & Anita, 1996) and similarly, some doctors and particularly pharmacists in this study have indicated choosing an injection was to avoid non-compliance problems.

### Limitations

The reliability of the study results involved a triangulation method, matching the prescription data from an earlier report with questionnaire responses from doctors, pharmacists and pharmacy technicians (Dorj et al., 2013). Despite the strengths of this study, some methodological aspects must be considered when interpreting results. Firstly, the selection of pharmacists and pharmacy technicians was based on a purposive selection of 40 community pharmacies. Also, the relatively small number of samples of health settings (eleven hospitals and 20 FGPs located in Ulaanbaatar city) may lead to selection bias and imprecise estimates. However, the high response rate of respondents in the questionnaire studies (pharmacists and pharmacy technicians (76%), doctors (89%)) was likely to avoid significant responder bias. In addition, the doctors were recruited randomly from the list provided by the human resource department of each hospital. This study recruited more specialists than general doctors, suggesting that the results may be more generalizable to them. However, the study included twenty-two general doctors, also providing information about their treatment of mild/moderate CAP patients.

In addition, the issue of compliance was only addressed in terms of whether it was an overall factor influencing the prescribing of injections over oral preparations which is the only dosage form recommended for adults. Compliance is a complex issue for children as an injectable product (gentamicin) is included in the STGs for children. From a compliance perspective for children, there is a range of factors influencing compliance dependent on the child’s age and parental involvement and whether it is compliant with the daily dosing schedule or the duration of the antibiotic or both. Hence, this was too complex an issue to address fully in this study.

## Conclusion

Non-compliance with STGs in respect to prescribing injections for mild/moderate CAP was evident in Mongolia. In addition, the high prevalence of the prescribing of non-antibiotic injections was inappropriate for mild/moderate CAP. The supply of antibiotic injections OTC from pharmacies, although currently indicating a similar range of selections being made to those prescribed by physicians, should be ceased unless this would markedly reduce access to treatment for poorer patients. Additional measures need to be put in place with the aim of reducing the high level of inappropriate injections prescribed in Mongolia. It is notable that the current standard treatment guidelines are followed by less than 40% of prescribers in Mongolia.

## Supplemental Information

10.7717/peerj.1375/supp-1 Supplemental Information 1 Raw data: doctors

10.7717/peerj.1375/supp-2 Supplemental Information 2 Raw data: pharmacists

10.7717/peerj.1375/supp-3 Supplemental Information 3 Data collection—doctors

10.7717/peerj.1375/supp-4 Supplemental Information 4 Data collection—pharmacists

## Additional Information and Declarations

### Data Availability

The following information was supplied regarding data availability: Raw data can be found in Supplemental Information and in Dryad: 10.5061/dryad.nm722.
