# Supplementary material for: A questionnaire study of injections prescribed and dispensed for patients diagnosed with mild/moderate community-acquired pneumonia in Mongolia
Source: PeerJ. 2015 Nov 26;3:e1375. doi: 10.7717/peerj.1375 (PMC4671187; doi:10.7717/peerj.1375)
Supplement: Supplemental Information 3 [file peerj-03-1375-s003.doc]

**Interview with doctors**

**DATA COLLECTION FORM**

_______________________

Date

Code _____________________

Residential location_____________________

1. Age:  20-30  31-50  51-60  61+
2. Gender:  M  F
3. Work level:  FGP  Public hospital  Private hospital  Others
4. Medical Role:  G/P  Specialist
5. Years of work in this field: _______
6. When prescribing antibiotics for patients with community-acquired pneumonia (CAP), what are the issues that influence your prescribing?

|  | **Never**  **0%** | **Rarely**  **1-10%** | **Sometimes**  **11-40%** | **Often**  **41-80%** | **Always**  **>80%** |
| --- | --- | --- | --- | --- | --- |
| 1. Patient expectations/demand |  |  |  |  |  |
| 1. Essential drug list with   reimbursement |  |  |  |  |  |
| 1. Drug company information |  |  |  |  |  |
| 1. Drug company representative visits |  |  |  |  |  |
| 1. Treatment guidelines for CAP |  |  |  |  |  |
| 1. Information from CPD programs/   seminars |  |  |  |  |  |
| 1. Likelihood of adverse effects |  |  |  |  |  |
| 1. Regional antibiotic sensitivity data |  |  |  |  |  |
| 1. Patient antibiotic sensitivity data |  |  |  |  |  |
| 1. Journals, publications, articles |  |  |  |  |  |
| 1. Influence of peers, fellow GP’s |  |  |  |  |  |
| 1. Influence of specialists |  |  |  |  |  |
| 1. Personal experience |  |  |  |  |  |
| 1. Information about previous use of   antibiotics obtained from a pharmacy by the patient |  |  |  |  |  |
| 1. Drug availability |  |  |  |  |  |
| 1. Affordability of medications for   patient |  |  |  |  |  |
| 1. Broad spectrum of antibiotic activity are the best option |  |  |  |  |  |
| 1. Preference for recently marketed   medications |  |  |  |  |  |
| 1. Government monitoring of   prescribing |  |  |  |  |  |
| 1. Risk of being charged for litigation |  |  |  |  |  |
| 1. Incentives from pharmaceutical   companies |  |  |  |  |  |

1. When prescribing a particular dosage form for the treatment of CAP, what issues influence that choice?

|  | **Never**  **0%** | **Rarely**  **1-10%** | **Sometimes**  **11-40%** | **Often**  **41-80%** | **Always**  **>80%** |
| --- | --- | --- | --- | --- | --- |
| 1. Injections are more effective than   oral administration |  |  |  |  |  |
| 1. Patients prefer an oral medication rather than treatment with injections |  |  |  |  |  |
| 1. The medication product quality is   better in an injection rather than tablet or capsule |  |  |  |  |  |
| 1. Adverse effects are less likely with an oral than injection treatment |  |  |  |  |  |
| 1. The treatment with oral medications is a more costly form of treatment than an injection including the cost of syringes, needles and the administration |  |  |  |  |  |
| 1. More repeat visits to the   hospital/clinic are caused by injections |  |  |  |  |  |
| 1. New needles, syringes and   single dose ampoules are  necessary for injections |  |  |  |  |  |
| 1. To switch from injection to oral administration during an antibiotic course for CAP |  |  |  |  |  |
| 1. Drug companies promote   injectable rather than oral medications |  |  |  |  |  |
| 1. Injections are chosen to provide better patient compliance |  |  |  |  |  |
| 1. Your medical training promoted the use of injections rather than oral medication |  |  |  |  |  |
| 1. The severity of CAP influences the prescribing of injections |  |  |  |  |  |
| 1. Patient demographic characteristics have an influence on the prescribing |  |  |  |  |  |

1. The normal duration of prescribing antibiotics for CAP by injection is:

≤3 days  4-5 days  > 5 days

1. The normal duration of prescribing antibiotics for CAP orally is:

≤ 3 days  4-5 days  > 5 days

1. If you switch a patient with CAP from injection to oral when do you recommend that the oral dosage starts:

≤ 24 hours  2 days  3-5 days  > 5 days after the initial treatment

1. Do you find the Mongolian treatment guidelines for CAP appropriate?

Yes  No  Don’t know

1. Do you prescribe more than one antibiotic for CAP at the same time?

|  | **Never**  **0%** | **Rarely**  **1-10%** | **Sometimes**  **11-40%** | **Often**  **41-80%** | **Always**  **>80%** |
| --- | --- | --- | --- | --- | --- |
|  |  |  |  |  |  |

1. How often do you have to change the antibiotic as the first one did not work?

|  | **Never**  **0%** | **Rarely**  **1-10%** | **Sometimes**  **11-40%** | **Often**  **41-80%** | **Always**  **>80%** |
| --- | --- | --- | --- | --- | --- |
|  |  |  |  |  |  |

1. List of antibiotics that you frequently prescribe for CAP

|  |  | **Never**  **0%** | **Rarely**  **1-10%** | **Sometimes**  **11-40%** | **Often**  **41-80%** | **Always**  **>80%** |
| --- | --- | --- | --- | --- | --- | --- |
|  | Penicillin, oral |  |  |  |  |  |
|  | Penicillin, injection |  |  |  |  |  |
|  | Amoxicillin, oral |  |  |  |  |  |
|  | Amoxicillin, injection |  |  |  |  |  |
|  | Ampicillin, oral |  |  |  |  |  |
|  | Ampicillin, injection |  |  |  |  |  |
|  | Ciprofloxacin, oral |  |  |  |  |  |
|  | Ciprofloxacin, injection |  |  |  |  |  |
|  | Cefazolin, oral |  |  |  |  |  |
|  | Cefazolin, injection |  |  |  |  |  |
|  | Erythromycin, oral |  |  |  |  |  |
|  | Erythromycin, injection |  |  |  |  |  |
|  | Amoxicillin/clavulanate, oral |  |  |  |  |  |
|  | Clarythromycin, oral |  |  |  |  |  |
|  | Clarythromycin, injection |  |  |  |  |  |
|  | Azithromycin, oral |  |  |  |  |  |
|  | Azithromycin, injection |  |  |  |  |  |
|  | Levofloxacin, oral |  |  |  |  |  |
|  | Tetracycline, oral |  |  |  |  |  |
|  | Trimethopim- sulfamethoxazole, oral |  |  |  |  |  |
|  | Doxycycline, oral |  |  |  |  |  |

1. What other medication group would you prescribe with antibiotics for CAP?

|  |  | **Never**  **0%** | **Rarely**  **1-10%** | **Sometimes**  **11-40%** | **Often**  **41-80%** | **Always**  **>80%** |
| --- | --- | --- | --- | --- | --- | --- |
|  | Dexamethasone, oral |  |  |  |  |  |
|  | Dexamethasone, injection |  |  |  |  |  |
|  | Bromhexine, oral |  |  |  |  |  |
|  | Acidi ascorbinici, oral |  |  |  |  |  |
|  | Acidi ascorbinici, injection |  |  |  |  |  |
|  | Chlorfenamin, tab |  |  |  |  |  |
|  | Vitamin B complex, oral |  |  |  |  |  |
|  | Vitamin B complex, injection |  |  |  |  |  |
|  | Cocorcarboxylase, injection |  |  |  |  |  |
|  | Euphyllin, oral |  |  |  |  |  |
|  | Euphyllin, injection |  |  |  |  |  |
|  | Analgin, oral |  |  |  |  |  |
|  | Analgin, injection |  |  |  |  |  |
|  | Dimedrol, oral |  |  |  |  |  |
|  | Dimedrol, injection |  |  |  |  |  |

1. How often do you receive governmental information about prescribing antibiotics?

|  | **Never** | **Weekly** | **Monthly** | **3 times a year** | **Once a year** |
| --- | --- | --- | --- | --- | --- |
|  |  |  |  |  |  |

1. To what extent do the patients come to you for treatment of CAP who have already purchased antibiotics from the following?

|  | **Never**  **0%** | **Rarely**  **1-10%** | **Sometimes**  **11-40%** | **Often**  **41-80%** | **Always**  **>80%** |
| --- | --- | --- | --- | --- | --- |
| - 1. Pharmacy |  |  |  |  |  |
| - 1. Market |  |  |  |  |  |
| - 1. Other, specify_____ |  |  |  |  |  |

1. When you prescribe antibiotics what is the frequency of generic prescribing?

|  | **Never**  **0%** | **Rarely**  **1-10%** | **Sometimes**  **11-40%** | **Often**  **41-80%** | **Always**  **>80%** |
| --- | --- | --- | --- | --- | --- |
|  |  |  |  |  |  |

1. Where do you obtain antibiotic sensitivity data from?

|  | **Never**  **0%** | **Rarely**  **1-10%** | **Sometimes**  **11-40%** | **Often**  **41-80%** | **Always**  **>80%** |
| --- | --- | --- | --- | --- | --- |
| 1. Governmental information |  |  |  |  |  |
| 1. Governmental publications |  |  |  |  |  |
| 1. Antibiotic package leaflet |  |  |  |  |  |
| 1. Hospital |  |  |  |  |  |
| 1. Treated patients |  |  |  |  |  |
| 1. Colleagues |  |  |  |  |  |
| 1. Antibiotics not working |  |  |  |  |  |
| 1. Internet |  |  |  |  |  |

1. How frequently do you admit/send a patient to hospital with CAP?

|  | **Never**  **0%** | **Rarely**  **1-10%** | **Sometimes**  **11-40%** | **Often**  **41-80%** | **Always**  **>80%** |
| --- | --- | --- | --- | --- | --- |
|  |  |  |  |  |  |

1. Do you think that injections for treatment of diseases in general are overused in Mongolia?

|  | **SA** | **A** | **D** | **SD** | **NR** |
| --- | --- | --- | --- | --- | --- |
|  |  |  |  |  |  |

1. If yes, please specify the reasons?

|  | **SA** | **A** | **D** | **SD** | **NR** |
| --- | --- | --- | --- | --- | --- |
| 1. Patients are able to easily buy the medicines from many pharmacies |  |  |  |  |  |
| 1. Lack of government control on drug sale |  |  |  |  |  |
| 1. Public demand |  |  |  |  |  |

1. Are you aware of counterfeit medicines in Mongolia?  Yes  No
2. If yes, have you experienced problems with counterfeit medicines?

|  | **Never**  **0%** | **Rarely**  **1-10%** | **Sometimes**  **11-40%** | **Often**  **41-80%** | **Always**  **>80%** |
| --- | --- | --- | --- | --- | --- |
| 1. Antibiotics |  |  |  |  |  |
| 1. Other medications |  |  |  |  |  |

1. May I ask about your approximate monthly income?

≤ 90.000MNT  91-200.000MNT 201-300.000MNT

301-400.000MNT  401-500.000MNT  ≥501.000MNT

1. Do you want to discuss about any other issues related to CAP and its treatment in Mongolia?

______________________________________________

**Thank you for your time**.
