# Supplementary material for: A questionnaire study of injections prescribed and dispensed for patients diagnosed with mild/moderate community-acquired pneumonia in Mongolia
Source: PeerJ. 2015 Nov 26;3:e1375. doi: 10.7717/peerj.1375 (PMC4671187; doi:10.7717/peerj.1375)
Supplement: Supplemental Information 4 [file peerj-03-1375-s004.doc]

**Interview with Pharmacists/pharmacy technicians**

**DATA COLLECTION FORM**

_______________________

Date

Code _____________________

Residential location_____________________

1. Age:  20-30  30-50  50-60  60+
2. Gender:  M  F
3. Working level:  Owner  Employee
4. Pharmaceutical role:  Pharmacist  Pharmacy technician
5. Years of work as pharmacist/pharmacy technician: ______________________

***The following questions are related to medicines that are prescribed.***

1. ***List the antibiotics that are being frequently dispensed for community-acquired pneumonia (CAP) with a prescription from a doctor***

|  |  | **Never**  **0%** | **Rarely**  **1-10%** | **Sometimes**  **11-40%** | **Often**  **41-80%** | **Always**  **>80%** |
| --- | --- | --- | --- | --- | --- | --- |
|  | Penicillin, oral |  |  |  |  |  |
|  | Penicillin, injection |  |  |  |  |  |
|  | Amoxicillin, oral |  |  |  |  |  |
|  | Amoxicillin, injection |  |  |  |  |  |
|  | Ampicillin, oral |  |  |  |  |  |
|  | Ampicillin, injection |  |  |  |  |  |
|  | Ciprofloxacin, oral |  |  |  |  |  |
|  | Ciprofloxacin, injection |  |  |  |  |  |
|  | Cefazolin, oral |  |  |  |  |  |
|  | Cefazolin, injection |  |  |  |  |  |
|  | Erythromycin, oral |  |  |  |  |  |
|  | Erythromycin, injection |  |  |  |  |  |
|  | Amoxicillin/clavulanate, oral |  |  |  |  |  |
|  | Clarythromycin, oral |  |  |  |  |  |
|  | Clarythromycin, injection |  |  |  |  |  |
|  | Azithromycin, oral |  |  |  |  |  |
|  | Azithromycin, injection |  |  |  |  |  |
|  | Levofloxacin, oral |  |  |  |  |  |
|  | Tetracycline, oral |  |  |  |  |  |
|  | Trimethopim- sulfamethoxazole, oral |  |  |  |  |  |
|  | Doxycycline, oral |  |  |  |  |  |

1. What other prescribed medications are also prescribed with antibiotics for CAP?

|  |  | **Never**  **0%** | **Rarely**  **1-10%** | **Sometimes**  **11-40%** | **Often**  **41-80%** | **Always**  **>80%** |
| --- | --- | --- | --- | --- | --- | --- |
|  | Dexamethasone, oral |  |  |  |  |  |
|  | Dexamethasone, injection |  |  |  |  |  |
|  | Bromhexine, oral |  |  |  |  |  |
|  | Acidi ascorbinici, oral |  |  |  |  |  |
|  | Acidi ascorbinici, injection |  |  |  |  |  |
|  | Chlorfenamin, tab |  |  |  |  |  |
|  | Vitamin B complex, oral |  |  |  |  |  |
|  | Vitamin B complex, injection |  |  |  |  |  |
|  | Cocorcarboxylase, injection |  |  |  |  |  |
|  | Euphyllin, oral |  |  |  |  |  |
|  | Euphyllin, injection |  |  |  |  |  |
|  | Analgin, oral |  |  |  |  |  |
|  | Analgin, injection |  |  |  |  |  |
|  | Dimedrol, oral |  |  |  |  |  |
|  | Dimedrol, injection |  |  |  |  |  |

1. How frequently do the doctors prescribe more than one antibiotic for patients with CAP at the same time?

|  | **Never**  **0%** | **Rarely**  **1-10%** | **Sometimes**  **11-40%** | **Often**  **41-80%** | **Always**  **>80%** |
| --- | --- | --- | --- | --- | --- |
|  |  |  |  |  |  |

1. When dispensing a particular dosage form that is prescribed by a doctor for patients with CAP, what are issues that influence your dispensing?

*SA: Strongly agree, A: Agree, D: Disagree, SD: Strongly Disagree, NR: No response*

|  | **SA** | **A** | **D** | **SD** | **NR** |
| --- | --- | --- | --- | --- | --- |
| 1. Essential drug list with reimbursement |  |  |  |  |  |
| 1. Medical profile of children |  |  |  |  |  |
| 1. Medical profile of adults |  |  |  |  |  |
| 1. Patient characteristics, severity |  |  |  |  |  |
| 1. Dosage forms of the prescribed medicine |  |  |  |  |  |
| 1. Duration of the prescribed medications |  |  |  |  |  |
| 1. Knowledge about adverse reactions, side effects |  |  |  |  |  |
| 1. Medical- legal concerns |  |  |  |  |  |
| 1. Treatment guideline information |  |  |  |  |  |
| 1. Patient compliance with medications |  |  |  |  |  |
| 1. Patient is not satisfied if not injected |  |  |  |  |  |
| 1. Affordability of medications to the patient |  |  |  |  |  |
| 1. Cost of brand vs generic medicines is important when dispensing |  |  |  |  |  |
| 1. Expiry date of medication |  |  |  |  |  |
| 1. Need for reconstitution |  |  |  |  |  |

1. How frequently do you have to change the prescriptions for CAP because the prescription

appears to be inappropriate?

|  | **Never**  **0%** | **Rarely**  **1-10%** | **Sometimes**  **11-40%** | **Often**  **41-80%** | **Always**  **>80%** |
| --- | --- | --- | --- | --- | --- |
|  |  |  |  |  |  |

1. The normal duration of **prescribed** antibiotics for CAP by injection is:

≤3 days  4-5 days  > 5 days

1. The normal duration of **prescribed** antibiotics for CAP orally is:

≤ 3 days  4-5 days  > 5 days

1. If the treatment of CAP is switched from injection to oral, the time of the switch from an injection is:

≤ 24 hours  2 days  3 days  > 5 days after commencing treatment

***The following questions are related to medicines that are***

***dispensed in the pharmacy without prescription.***

1. List the antibiotics that are being frequently dispensed for community-acquired pneumonia

***(CAP) without a prescription***

|  |  | **Never**  **0%** | **Rarely**  **1-10%** | **Sometimes**  **11-40%** | **Often**  **41-80%** | **Always**  **>80%** |
| --- | --- | --- | --- | --- | --- | --- |
|  | Penicillin, oral |  |  |  |  |  |
|  | Penicillin, injection |  |  |  |  |  |
|  | Amoxicillin, oral |  |  |  |  |  |
|  | Amoxicillin, injection |  |  |  |  |  |
|  | Ampicillin, oral |  |  |  |  |  |
|  | Ampicillin, injection |  |  |  |  |  |
|  | Ciprofloxacin, oral |  |  |  |  |  |
|  | Ciprofloxacin, injection |  |  |  |  |  |
|  | Cefazolin, oral |  |  |  |  |  |
|  | Cefazolin, injection |  |  |  |  |  |
|  | Erythromycin, oral |  |  |  |  |  |
|  | Erythromycin, injection |  |  |  |  |  |
|  | Amoxicillin/clavulanate, oral |  |  |  |  |  |
|  | Clarythromycin, oral |  |  |  |  |  |
|  | Clarythromycin, injection |  |  |  |  |  |
|  | Azithromycin, oral |  |  |  |  |  |
|  | Azithromycin, injection |  |  |  |  |  |
|  | Levofloxacin, oral |  |  |  |  |  |
|  | Tetracycline, oral |  |  |  |  |  |
|  | Trimethopim- sulfamethoxazole, oral |  |  |  |  |  |
|  | Doxycycline, oral |  |  |  |  |  |

1. What other medications would you dispense with antibiotics for CAP without a prescription?

|  |  | **Never**  **0%** | **Rarely**  **1-10%** | **Sometimes**  **11-40%** | **Often**  **41-80%** | **Always**  **>80%** |
| --- | --- | --- | --- | --- | --- | --- |
|  | Dexamethasone, oral |  |  |  |  |  |
|  | Dexamethasone, injection |  |  |  |  |  |
|  | Bromhexine, oral |  |  |  |  |  |
|  | Acidi ascorbinici, oral |  |  |  |  |  |
|  | Acidi ascorbinici, injection |  |  |  |  |  |
|  | Chlorfenamin, tab |  |  |  |  |  |
|  | Vitamin B complex, oral |  |  |  |  |  |
|  | Vitamin B complex, injection |  |  |  |  |  |
|  | Cocorcarboxylase, injection |  |  |  |  |  |
|  | Euphyllin, oral |  |  |  |  |  |
|  | Euphyllin, injection |  |  |  |  |  |
|  | Analgin, oral |  |  |  |  |  |
|  | Analgin, injection |  |  |  |  |  |
|  | Dimedrol, oral |  |  |  |  |  |
|  | Dimedrol, injection |  |  |  |  |  |

1. When dispensing a particular dosage form for the treatment of CAP without a prescription, what issues influence that choice?

|  | **SA** | **A** | **D** | **SD** | **NR** |
| --- | --- | --- | --- | --- | --- |
| 1. Injections are more effective than oral   administration |  |  |  |  |  |
| 1. The medication product quality is better in an injection rather than tablet or capsule |  |  |  |  |  |
| 1. Adverse effects are less likely with an oral than injection treatment |  |  |  |  |  |
| 1. The doses of injections are chosen to provide better patient compliance |  |  |  |  |  |
| 1. New needles, syringes and single dose ampoules are necessary for injections |  |  |  |  |  |
| 1. There is no treatment benefit to switch from injection to oral during an antibiotic course for CAP |  |  |  |  |  |
| 1. Your pharmaceutical training promoted the use of injections rather than oral medication |  |  |  |  |  |
| 1. Drug companies promote injectable rather than oral medications |  |  |  |  |  |
| 1. Prefer to dispense newly marketed products |  |  |  |  |  |
| 1. The total treatment with oral medications is a more costly form of treatment than with injections including the cost of syringes, needles and administration |  |  |  |  |  |
| 1. More repeat visits to the pharmacies are caused by injections |  |  |  |  |  |
| 1. Injections are chosen to provide better patient compliance |  |  |  |  |  |
| 1. Patients prefer an oral medication rather than treatment with injections |  |  |  |  |  |
| 1. The age and gender of the patients can have influence on dispensing injections |  |  |  |  |  |
| 1. The severity of the patient with CAP influences the dispensing of injections |  |  |  |  |  |

1. Do you dispense more than one antibiotic without prescription for CAP at the same time?

|  | **Never**  **0%** | **Rarely**  **1-10%** | **Sometimes**  **11-40%** | **Often**  **41-80%** | **Always**  **>80%** |
| --- | --- | --- | --- | --- | --- |
|  |  |  |  |  |  |

1. The normal duration of **dispensed** antibiotics for CAP by injection is:

≤3 days  4-5 days  > 5 days

1. The normal duration of **dispensed** antibiotics for CAP orally is:

≤ 3 days  4-5 days  > 5 days

1. If the treatment of CAP is switched from injection to oral, the time of the switch from an injection is:

≤ 24 hours  2 days  3 days  > 5 days after commencing treatment

1. How often do you receive governmental information about antibiotic sensitivity data?

|  | **Never** | **Weekly** | **Monthly** | **3 times a year** | **Once a year** |
| --- | --- | --- | --- | --- | --- |
|  |  |  |  |  |  |

1. Do you find the current Mongolian treatment guidelines for CAP appropriate?

Yes  No  NR

1. How often would you refer a patient with CAP who comes to the pharmacy to a doctor?

|  | **Never**  **0%** | **Rarely**  **1-10%** | **Sometimes**  **11-40%** | **Often**  **41-80%** | **Always**  **>80%** |
| --- | --- | --- | --- | --- | --- |
|  |  |  |  |  |  |

1. Do you consider injections s more effective treatment for CAP?  Yes  No
2. If yes, what is the effect of injections?

|  | **Never**  **0%** | **Rarely**  **1-10%** | **Sometimes**  **11-40%** | **Often**  **41-80%** | **Always**  **>80%** |
| --- | --- | --- | --- | --- | --- |
| 1. More rapid cure |  |  |  |  |  |
| 1. Adverse effects are less frequent than with oral treatment |  |  |  |  |  |

1. To what extent do you agree that there is more financial benefit with injections to the following people?

|  | **Never**  **0%** | **Rarely**  **1-10%** | **Sometimes**  **11-40%** | **Often**  **41-80%** | **Always**  **>80%** |
| --- | --- | --- | --- | --- | --- |
| 1. Doctor |  |  |  |  |  |
| 1. Pharmacist |  |  |  |  |  |
| 1. Patient |  |  |  |  |  |
| 1. Nurse |  |  |  |  |  |

1. Do you charge a special fee for administering injections?

Yes, amount_______  No

1. Do you think the fee for dispensing and administering injections is affordable to the patient?

Yes  No  NR

1. When dispensing injections, which of the following are considered:

|  | **Never**  **0%** | **Rarely**  **1-10%** | **Sometimes**  **11-40%** | **Often**  **41-80%** | **Always**  **>80%** |
| --- | --- | --- | --- | --- | --- |
| 1. Supplied from reliable source |  |  |  |  |  |
| 1. Using sterile drips, syringes and needles |  |  |  |  |  |
| 1. Package condition of the medication |  |  |  |  |  |
| 1. Patient’s self diagnosis and request for injection |  |  |  |  |  |
| 1. Reconstitution of the antibiotic |  |  |  |  |  |
| 1. Expiry date of the reconstituted product |  |  |  |  |  |

1. Do you think that injections for treatment of diseases in general are overused in Mongolia?

|  | **SA** | **A** | **D** | **SD** | **NR** |
| --- | --- | --- | --- | --- | --- |
|  |  |  |  |  |  |

1. If yes, please specify the reasons?

|  | **SA** | **A** | **D** | **SD** | **NR** |
| --- | --- | --- | --- | --- | --- |
| 1. Patients are able to easily buy injections from many pharmacies |  |  |  |  |  |
| 1. Lack of government control on drug sale |  |  |  |  |  |
| 1. Public demand for injections is high |  |  |  |  |  |

1. After using a disposable syringe:

|  | **Never**  **0%** | **Rarely**  **1-10%** | **Sometimes**  **11-40%** | **Often**  **41-80%** | **Always**  **>80%** |
| --- | --- | --- | --- | --- | --- |
| 1. You change the needle and retain the syringe for reuse |  |  |  |  |  |
| 1. You sterilize the syringe and needle and reuse it |  |  |  |  |  |
| 1. You discard all |  |  |  |  |  |
| 1. You discard and destroy it after the first time it was used |  |  |  |  |  |

1. When administering an intravenous drip:

|  | **Never**  **0%** | **Rarely**  **1-10%** | **Sometimes**  **11-40%** | **Often**  **41-80%** | **Always**  **>80%** |
| --- | --- | --- | --- | --- | --- |
| 1. You give the whole vial as a drip to a patient |  |  |  |  |  |
| 1. You retain the residual not required for that dose |  |  |  |  |  |
| 1. You reconstitute what remained of the powder for the next patient |  |  |  |  |  |
| 1. You discard everything the first time you used it |  |  |  |  |  |

1. From where do you obtain injectable drugs for the treatment of diseases?

|  | **Never**  **0%** | **Rarely**  **1-10%** | **Sometimes**  **11-40%** | **Often**  **41-80%** | **Always**  **>80%** |
| --- | --- | --- | --- | --- | --- |
| 1. Pharmaceutical wholesaler |  |  |  |  |  |
| 1. Pharmacy |  |  |  |  |  |
| 1. Detailer |  |  |  |  |  |
| 1. Others (private import) |  |  |  |  |  |

1. Are you aware of counterfeit medicines in Mongolia?  Yes  No
2. If yes, have you experienced problems with counterfeit medicines?

|  | **Never**  **0%** | **Rarely**  **1-10%** | **Sometimes**  **11-40%** | **Often**  **41-80%** | **Always**  **>80%** |
| --- | --- | --- | --- | --- | --- |
| - 1. Antibiotics |  |  |  |  |  |
| - 1. Other medications |  |  |  |  |  |

1. May I ask about your approximate monthly income?

≤ 90.000MNT  91-200.000MNT 201-300.000MNT

301-400.000MNT  401-500.000MNT  ≥501.000MNT

1. Do you want to discuss any other issues related to prescribing for CAP and its treatment in Mongolia?

**Thank you for your time.**
